# The extent and determinants of diabetes and cardiovascular disease comorbidity in South Africa – results from the South African National Health and Nutrition Examination Survey (SANHANES-1)

**DOI:** 10.1186/s12889-017-4792-8

**Published:** 2017-09-26

**Authors:** Chipo Mutyambizi, Lumbwe Chola, Wim Groot, Milena Pavlova, Demetre Labadarios, Charles Hongoro

**Affiliations:** 1 Population Health, Health Systems and Innovation, Human Sciences Research Council, HSRC Building, 134 Pretorius Street, Pretoria, 0002 South Africa; 2 School of Public Health, Faculty of Health Sciences, University of the Witwatersrand, Johannesburg, South Africa; 3 Department of Health Services Research, CAPHRI, Maastricht University Medical Centre, Faculty of Health, Medicine and Life Sciences, Maastricht University, Maastricht, The Netherlands

**Keywords:** Diabetes, Comorbidity, South Africa, Social determinants

## Abstract

**Background:**

Diabetes is a major health problem and cause of death worldwide. It is predicted that the prevalence of diabetes will increase from 415 million in 2015 to 642 million in 2040. However, the burden of diabetes in low- and middle-income countries is not clearly understood, particularly its interaction with other chronic illnesses. This study investigates the self-reported prevalence of and factors associated with diabetes and cardiovascular comorbidity in South Africa.

**Methods:**

Data used in this study are from the 2012 South African National Health and Nutrition Examination Survey; a nationally representative cross-sectional household survey (*N* = 25,532). Diabetes and cardiovascular disease comorbidity was defined as the coexistence of diabetes plus one or more cardiovascular diseases reported at the time of the survey. This study makes use of multinomial logistic regression models to analyse the relationship between diabetes - cardiovascular disease comorbidity and several predictors including race, income, socio-economic status and obesity.

**Results:**

According to the survey data we analysed, 5% of South Africans aged 15 and above had self-reported diabetes in 2011–2012. Among those with self-reported diabetes, 73% had at least one additional cardiovascular chronic illness. Diabetes and its cardiovascular disease comorbidity was more prevalent in Africans (66%), females (66%), those who lived in urban areas (75%), had secondary education (44%) and were unemployed (62%). Factors strongly associated with diabetes - cardiovascular disease comorbidity were older age (Odds ratio [OR] 1.09; 95% Confidence Interval [CI] 1.06–1.12), high household income (0.27; 0.10–0.76) versus low income, moderate (0.33; 0.11–0.96) and good self-rated health (0.24; 0.08–0.68) versus bad self-rated health, occasional (0.29; 0.10–0.88) and regular smokers (0.25; 0.12–0.53) versus non-smokers and physical activity (0.15; 0.03–0.68) versus no physical activity.

**Conclusion:**

The study provides insight into the factors associated with cardiovascular disease comorbidity in diabetic individuals. The findings indicate that there are differences in the factors associated with diabetes and those associated with diabetes - cardiovascular disease comorbidity. This provides information, which can be used to design programmes that encourage healthy lifestyles in people living with diabetes.

## Background

Diabetes is a chronic illness that is fast becoming a world pandemic affecting one in 11 adults in 2015 [1]. Globally, the prevalence of diabetes is expected to increase from 415 million in 2015 to 642 million in 2040 [1]. More rapid increases are expected in low- and middle-income countries (LMICs) [2]. Although the prevalence of both type 1 and type 2 diabetes is increasing all over the world, type 2 diabetes is reported to be the most common type of diabetes globally [1]. Diabetes and its related illnesses is an increasing cause of premature death and it is estimated to have caused significantly more deaths in 2015 than in 2005. Mortality from diabetes increased by 32.1% in the period 2005 to 2015 (1.5 million deaths globally in 2015) whilst mortality from diabetic kidney disease increased by 39.5% in the same period (418,000 deaths globally in 2015) [3]. In South Africa, diabetes ranked third amongst the top 10 causes of death in 2014 [4]. The statistics of the rising prevalence and mortality due to diabetes globally makes it one of the biggest public health concerns of the twenty-first century [1].

Along with an increased prevalence of diabetes as a single cause of morbidity, there is increasing evidence that individuals with diabetes experience one or more micro- or macro-vascular complications, including but not limited to cardiovascular diseases (CVDs), blindness, peripheral neuropathy and kidney disease [5]. Individuals with diabetes are at an increased risk of CVDs such as stroke, heart attack and heart failure when compared to individuals without diabetes [6] and the presence of high blood pressure contributes to increased risks of CVD complications [1]. In addition, heart disease and stroke account for the highest mortality rates in people with diabetes when compared to those without [5]. In Africa, delayed diagnosis often leads to the development of diabetic complications [7]. Heart disease is estimated to affect 20% of diabetic patients whilst approximately 50% of diabetics suffer from heart muscle diseases [7]. It is also estimated that renal failure is the leading cause of death amongst hospitalised diabetic patients and that diabetes is the leading cause of amputations in Africa [7]. This suggests that the analysis of diabetes comorbidity is a relevant research topic in African countries, including South Africa.

Comorbidity has various definitions [8] but it is usually referred to as the presence of one or more additional illnesses in an individual affected by the index disease under study [9]. The concepts of comorbidities and complications have often been used interchangeably in epidemiologic research [10]. Whilst complications occur after diagnosis of an index disease and are a consequence of the index disease, comorbidities can be diagnosed before or after diagnosis of the index disease and are not a consequence of the index disease [10]. In some cases, separating the two may be a complex task when a condition does not clearly fit the criteria of being a comorbidity or complication [10]. For example high blood pressure is a common complication of diabetes but may also occur in isolation. Therefore this study makes use of a very broad definition of comorbidity. In this study comorbidity refers to a situation where an individual who has diabetes, also has one or more CVDs such as heart disease, stroke and high blood pressure. Comorbid chronic conditions, such as CVDs, can increase the complexity of the disease burden in people with diabetes, directly worsening their health outcomes whilst also placing a heavy burden on healthcare systems [6].

This is of great concern in South Africa given the rise in mortality due to non-communicable diseases (NCDs) within the country [11]. Due to the anticipated increases in the prevalence of diabetes, research into its interactions with other conditions, such as CVDs, is becoming increasingly important. The occurrence and burden of two or more illnesses in one person has mostly been studied in high income countries whilst research in LMICs is relatively scarce [12]. There is limited evidence of chronic disease comorbidities in South Africa in the published literature, and to our knowledge, there is no study on diabetes-CVD comorbidity. Alaba and Chola (2013) showed that the prevalence of chronic disease multi-morbidity in South African adults was 4% of the adult population, with obesity, smoking and depression being among the major determinants [13]. However, their study did not specifically focus on diabetic individuals. In our study, we highlight the CVD comorbid conditions common among diabetics and examine the association of diabetic comorbidity with various other determinants of health. This is important given the increasing prevalence of diabetic complications and potential implications on the health system. An understanding of the factors associated with diabetes and its comorbid conditions will inform decision making and interventions targeted at preventing and managing diabetic comorbidity. Therefore this paper investigates the self-reported prevalence of and factors associated with diabetes and cardiovascular comorbidity in South Africa.

## Methods

### Data

Data were taken from the 2012 South African National Health and Nutrition Examination Survey (SANHANES-1). This nationally representative cross-sectional household survey was conducted in April to November 2012 by the Human Sciences Research Council (HSRC) and investigated the health status of the South African population providing information on NCDs. The SANHANES-1 survey received clearance from the Research Ethics Committee (REC) of the HSRC (REC 6/16/11/11). Each respondent provided written consent prior to the interview. For those under the age of 18, authorised written consent was obtained from parents or guardians. The interviews were conducted by trained interviewers in the respondents’ homes and in the respondents’ preferred language. The survey applied a stratified, multi-stage cluster sample approach. The 2001 population census was used to select a total of 1000 census enumeration areas (EAs) that were stratified by locality type and province. A total of 500 EAs were then selected from the 1000 EAs yielding a sample that was representative of the socio-demographic profile of South Africa. A sample of 20 visiting points (VPs) was randomly selected from the EAs. This yielded a sample of 10,000 households. Out of the 10,000 households, 8166 were valid, occupied households, of which 77.2% agreed to participate in the survey. This resulted in a total of 27,580 eligible individuals (household members), of which 92.6% participated in the survey. A detailed report of the SANHANES-1 survey methodology is provided elsewhere [14].

### Measures

In the SANHANES-1 survey, the adult questionnaire provided information on diabetes and various other CVD conditions. Respondents were asked to state whether or not a physician or nurse or health worker at a clinic or hospital had told the respondents that they have or have had diabetes or any of the following CVD conditions; high blood pressure, stroke and heart disease. As such the diabetic comorbidities considered in this study are heart disease, high blood pressure and stroke. The prevalence of these illnesses was estimated using self-reported previously diagnosed health conditions. The SANHANES questionnaire did not ask respondents to specify the type of diabetes they had. In the analysis, these health conditions were used to create a categorical dependent variable of comorbidity ranging from 0 to 2. The categorical variable took on the following values; 0 = respondent did not have diabetes or any CVD, 1 = presence of diabetes without CVD comorbidity, 2 = presence of diabetes with CVD comorbidity (i.e. presence of diabetes and one or more of the chronic diseases mentioned above at the time of the survey).

The selection of independent variables was guided by the Commission on Social Determinants of Health (CSDH) model [15]. The model distinguishes structural and intermediary determinants of health. The structural determinants refer to the mechanisms that generate stratification in the society which in turn influences or defines individual social economic position [15]. The CSDH framework postulates that these structural determinants configure health opportunities based on economic status. Structural determinants include factors such as education, income, socio-economic position, and other social stratifiers, such as gender, ethnicity and age. The intermediary determinants include behavioural factors, psychosocial factors, biological factors and material circumstances such as living conditions. These intermediary determinants act as a pathway through which socio-economic position influences health. The framework posits that differences in material circumstances such as living standards result in health inequalities. Economically deprived individuals are exposed to health-compromising conditions which result in them more frequently experiencing worse health outcomes than the more privileged individuals.

Based on the CSDH model and the data available in our dataset, the structural variables in our model included gender, age, residence, race, household income, education and employment status. Age was measured in years (from 15 years) and was included as a continuous variable. Place of residence was included as a dichotomous variable with 0 – urban and 1 – rural. The SANHANES collected ethnicity data on the categories of African, white, coloured, Indian/Asian and ‘other’. These categories were based on the predominant ethnic groups in the 2001 census and are commonly included in analysis to assess inequalities due to the legacy of the pre 1994 racial hierarchy that existed in the country. None of the respondents declared ‘other’, therefore race was included as a categorical variable with 1 – African, 2 – White, 3 – Coloured and 4 – Indian/Asian. The SANHANES-1 survey collected annual income data by asking each adult household member which income category best described their individual gross annual income. We then calculated the midpoint estimate of each category and used this to derive the total gross annual income per household, which was then divided by the number of adults in each household in order to get average income per household. This was then re-categorised into quantiles ranging from low to high income. Education was measured in years of schooling and was categorised as 0 - no education, 1 - primary (1–7 years), 2 – secondary (8–12 years) and 3 – tertiary (13+ years). Employment was included as a categorical variable; 0 - unemployed, 1 – informal employment and 2 – formal employment.

The intermediary variables included body mass index (BMI), self-rated health, smoking status, alcohol consumption and physical activity. BMI was a continuous variable calculated as weight divided by height squared. It was then recoded into a categorical variable as 0 - underweight (BMI < 18.5), 1 – normal weight (BMI > =18.5 < 25), 2 – overweight (BMI > = 25 < 30) and 3 – obese (BMI ≥ 30). Self-rated health was included as a categorical variable as 0 – bad, 1 – moderate and 2 – good. Respondents in the SANHANES-1 survey were asked if they smoked tobacco. This was categorised into 0 – never, 1 – occasional and 2 – regularly. Alcohol consumption was also included as a categorical variable with 0 - never, 1 – occasional and 2 – regularly. Respondents were asked whether they took part in some form of vigorous intensity sport, fitness or recreational activities such as running or weight lifting that cause large increases in the heart rate for at least 10 min at a time. This was included as a binary variable 0 – no 1 – yes.

### Data analysis

In SANHANES-1 self-reported diabetes was only investigated in the adult questionnaire (individuals aged 15 and older). Observations with missing data on comorbidity were excluded (*n* = 6351). The final sample used in the analysis was *n* = 12,594. Statistical analyses were performed in STATA software version 14. In order to account for clustering and survey design effects, we used STATA’s stratified multi-stage design command. Therefore data was weighted to produce estimates that represent the country’s socio-demographic profile, based on the 2001 population census. For categorical variables, data was expressed as counts and proportions; mean values +/− standard deviations were reported for continuous variables. A multinomial logistic regression was conducted to analyse the association between several indicators such as social, economic, demographic and health variables, and having diabetes without CVD comorbidity, or diabetes with CVD comorbidity; the reference category was no diabetes or CVD. Step 1 of the regression analysis was the univariate or unadjusted analysis; step 2 was a multivariate analysis, which included structural variables only; step 3 was a multivariate analysis, which included intermediary variables only and the final step included all the variables in a multivariate analysis. In the analysis, the 95% confidence intervals (CI) and odds ratios (OR) are reported.

## Results

**Fig. 1:**
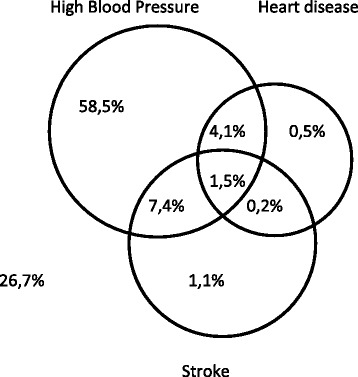
Cardiovascular comorbid conditions in diabetic individuals

**Fig. 2:**
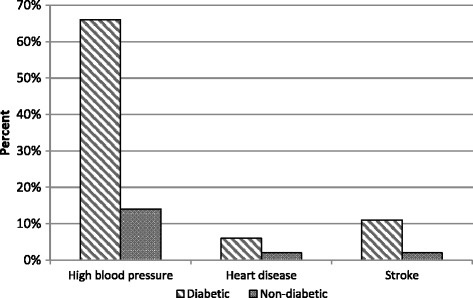
Prevalence of self-reported cardiovascular comorbidities among diabetic versus non-diabetic individuals

The prevalence of diabetes and CVD conditions is shown in Table 1. The sample consisted of 12,594 individuals of which 1000 (5%) reported having diabetes. Of the 1000 people who reported diabetes, approximately 73% had at least one of the CVD chronic illnesses included in the study, whilst 27% reported having diabetes and no CVD chronic illness included in the study. Approximately 2% of diabetic respondents reported having all three CVD chronic conditions; namely stroke, heart disease and high blood pressure (Fig. 1). The CVD comorbidities of high blood pressure, heart disease and stroke were more prevalent amongst diabetic individuals compared to non-diabetics (Fig. 2).

**Table 1:** Prevalence of self-reported diabetes and diabetic comorbidity (*N* = 12,594)

	None		Diabetes only		Diabetes + other	
Chronic Illnesses	(*N* = 11,594; 94%)		(*N* = 269; 2%)		(*N* = 731; 4%)	
Age: mean +/− sd	33	14.3	48	14.9	57	12.3
Gender						
Male	5163	50%	101	45%	225	34%
Female	6425	50%	168	55%	506	66%
Residence						
Urban	7676	67%	197	70%	551	75%
Rural	3918	33%	72	30%	180	25%
Race						
African	7873	79%	140	65%	387	66%
White	513	10%	18	17%	30	14%
Coloured	2230	8%	55	11%	176	13%
Indian	938	2%	56	7%	136	7%
Household income						
Low	3884	33%	82	31%	215	29%
Medium	4469	36%	105	34%	314	42%
High	3234	31%	82	35%	202	29%
Education						
None	628	5%	20	6%	78	10%
Primary	1588	13%	53	17%	186	26%
Secondary	6615	67%	132	63%	291	44%
Tertiary	1084	15%	28	14%	71	19%
Employment						
Unemployed	6752	58%	131	54%	395	62%
Informal	1201	10%	58	22%	165	17%
Formal	3393	32%	72	23%	148	21%
Obesity						
Underweight	462	3%	6	2%	14	1%
Normal Weight	2166	17%	29	12%	54	8%
Overweight	977	8%	41	16%	91	11%
Obese	7989	72%	193	69%	572	80%
Self-rated health						
Bad	414	3%	20	8%	111	16%
Moderate	1618	13%	80	32%	262	33%
Good	9341	83%	165	60%	348	50%
Smoking						
Never	317	15%	16	29%	62	53%
Occasional	188	9%	3	4%	11	4%
Regular	1907	76%	53	67%	85	43%
Alcohol						
Never	8442	72%	211	76%	603	80%
Occasional	2127	19%	43	23%	88	15%
Regular	782	9%	5	1%	31	4%
Physical Activity						
No	9775	84%	233	82%	682	96%
Yes	1618	16%	28	18%	36	4%

Note - Percentage sign (%) is mentioned where numbers and percentages are given; mean and standard deviation is mentioned for age, *n* number of observations, *sd* standard deviation. Values are weighted

As shown in Table 1, the majority of the respondents with diabetes-CVD comorbidity were female (66%). Most of the respondents with diabetes-CVD comorbidity lived in urban areas (75%), were African (66%), unemployed (62%), obese (80%), were in the medium income category (42%) and had secondary education (44%). About 43% were regular smokers, 4% consumed alcohol regularly, 16% reported having bad self-rated health and 96% did not take part in any vigorous physical activity. The mean age for diabetes-CVD comorbidity was 57 years (standard deviation – 12.3).

Table 2 and Table 3 show results from the multinomial logistic regression analysis, using those with no diabetes or CVD chronic illness as the reference category. Table 2 shows the results for the unadjusted and adjusted association of each variable with diabetes without CVD and Table 3 shows the results for the unadjusted and adjusted association of each variable with diabetes and CVD comorbidity (diabetes plus one or more CVD chronic illnesses). In step 1, the univariate model for respondents with diabetes without CVDs, age (Odds ratio [OR] 1.06; 95% Confidence Interval [CI]1.05–1.07), being coloured (1.68; 1.13–2.50) or Indian (3.65; 2.09–6.36) versus African, having informal employment (2.41; 1.25–4.66) versus unemployed were positively associated with having diabetes only, whilst good self-rated health (0.31; 0.17–0.56) versus bad self-rated health and regular alcohol consumption (0.11; 0.04–0.33) were negatively associated with having diabetes only, with statistically significant associations. In the univariate model for patients with diabetes and CVD comorbidity, all variables except income were significantly associated with this comorbidity.

**Table 2:** Logistic regression analyses of factors associated with diabetes without CVD comorbidity, no diabetes or CVD is the reference category

	Step 1-Unadjusted		Step 2-Structural		Step 3-Intermediary		Step 4-Full	
Diabetes Only	OR	(95% CI)	OR	(95% CI)	OR	(95% CI)	OR	(95% CI)
Age	1.06**	(1.05–1.07)	1.06**	(1.04–1.07)			1.06**	(1.03–1.08)
Gender								
Male	1		1				1	
Female	1.22	(0.81–1.85)	1.07	(0.63–1.83)			0.8	(0.34–1.84)
Residence								
Urban	1		1				1	
Rural	0.88	(0.61–1.27)	0.98	(0.63–1.53)			1	(0.32–3.00)
Race								
African	1		1				1	
White	2.05	(0.92–4.60)	1.29	(0.45–3.66)			1.05	(0.20–5.36)
Coloured	1.68**	(1.13–2.50)	1.61	(0.99–2.62)			1.58	(0.64–3.95)
Indian	3.65**	(2.09–6.36)	2.93**	(1.57–5.49)			3.49*	(1.11–10.96)
Household income								
Low	1		1				1	
Medium	0.99	(0.66–1.51)	0.73	(0.46–1.15)			0.93	(0.36–2.40)
High	1.19	(0.71–2.02)	0.78	(0.45–1.35)			0.47	(0.16–1.32)
Education								
None	1		1				1	
Primary	0.97	(0.51–1.83)	1.76	(0.87–3.53)			1.91	(0.36–10.14)
Secondary	0.70	(0.38–1.27)	2.39**	(1.18–4.84)			4.51	(0.77–26.47)
Tertiary	0.71	(0.34–1.51)	1.65	(0.51–5.30)			5.57	(0.70–44.97)
Employment								
Unemployed	1		1				1	
Informal	2.41**	(1.25–4.66)	1.41	(0.68–2.90)			0.79	(0.31–1.98)
Formal	0.79	(0.53–1.17)	0.71	(0.44–1.13)			0.90	(0.36–2.21)
Obesity								
Underweight	1				1			
Normal Weight	0.96	(0.30–3.10)			0.65	(0.13–3.39)	0.42	(0.07–2.67)
Overweight	2.84	(0.91–8.90)			2.63	(0.46–14.97)	0.90	(0.12–6.44)
Obese	1.29	(0.44–3.80)			0.59	(0.13–2.67)	0.35	(0.06–2.00)
Self-rated health								
Bad	1				1		1	
Moderate	1.02	(0.50–2.09)			1.04	(0.28–3.94)	1.34	(0.32–5.49)
Good	0.31**	(0.17–0.56)			0.52	(0.14–1.92)	0.79	(0.17–3.60)
Smoking								
Never	1				1		1	
Occasional	0.27	(0.04–1.84)			0.04**	(0.01–0.22)	0.03**	(0.00–0.26)
Regular	0.47	(0.19–1.13)			0.72	(0.32–1.61)	1.02	(0.40–2.65)
Alcohol								
Never	1				1		1	
Occasional	1.13	(0.60–2.11)			0.27**	(0.13–0.57)	0.31**	(0.13–0.76)
Regular	0.11**	(0.04–0.33)			0.04**	(0.01–0.24)	0.05**	(0.01–0.36)
Physical Activity								
No	1				1		1	
Yes	1.18	(0.55–2.53)			0.93	(0.35–2.46)	1.62	(0.52–5.05)

**p < 0.001; *p < 0.05; *OR* Odds ratio; 95%; *CI* Confidence Interval, *CVD* Cardiovascular disease

**Table 3:** Logistic regression analyses of factors associated with diabetes-CVD comorbidity, no diabetes or CVD is the reference category

	Step 1-Unadjusted		Step 2-Structural		Step 3-Intermediary		Step 4-Full	
Diabetes with CVDs comorbidity	OR	(95% CI)	OR	(95% CI)	OR	(95% CI)	OR	(95% CI)
Age	1.09**	(1.09–1.10)	1.1**	(1.09–1.11)			1.09**	(1.06–1.12)
Gender								
Male	1		1				1	
Female	1.96**	(1.49–2.59)	2.03**	(1.44–2.86)			1.43	(0.76–2.68)
Residence								
Urban	1		1				1	
Rural	0.68**	(0.53–0.86)	0.59**	(0.42–0.81)			0.44	(0.18–1.06)
Race								
African	1		1				1	
White	1.71	(0.85–3.42)	0.56	(0.26–1.23)			1.62	(0.54–4.84)
Coloured	1.82**	(1.45–2.29)	1.38*	(1.01–1.89)			1.94	(0.92–4.11)
Indian	3.74**	(2.57–5.45)	2.15**	(1.25–3.70)			2.76	(0.92–8.31)
Household income								
Low	1		1				1	
Medium	1.3	(0.90–1.89)	0.95	(0.63–1.43)			0.58	(0.28–1.17)
High	1.07	(0.71–1.60)	0.73	(0.39–1.36)			0.27*	(0.10–0.76)
Education								
None	1		1				1	
Primary	0.91	(0.62–1.34)	2.4**	(1.40–4.12)			1.17	(0.40–3.48)
Secondary	0.30**	(0.20–0.43)	2.32**	(1.29–4.18)			2.55	(0.78–8.33)
Tertiary	0.57	(0.29–1.12)	3.52**	(1.55–7.99)			3.33	(0.79–14.13)
Employment								
Unemployed	1		1				1	
Informal	1.67**	(1.21–2.30)	0.94	(0.62–1.43)			0.86	(0.30–2.48)
Formal	0.62**	(0.45–0.85)	0.87	(0.58–1.30)			0.71	(0.34–1.51)
Obesity								
Underweight	1				1		1	
Normal Weight	1.15	(0.51–2.60)			1.40	(0.29–6.73)	1.17	(0.17–7.87)
Overweight	3.62**	(1.68–7.80)			5.76*	(1.30–25.61)	3.08	(0.49–19.23)
Obese	2.85**	(1.37–5.93)			5.09*	(1.24–20.82)	3.27	(0.59–18.17)
Self-rated health								
Bad	1				1		1	
Moderate	0.52**	(0.37–0.75)			0.23	(0.10–0.54)	0.33*	(0.11–0.96)
Good	0.13**	(0.09–0.19)			0.15**	(0.07–0.36)	0.24**	(0.08–0.68)
Smoking								
Never	1				1		1	
Occasional	0.14**	(0.05–0.40)			0.18**	(0.06–0.52)	0.29*	(0.10–0.88)
Regular	0.16**	(0.07–0.36)			0.21**	(0.10–0.46)	0.25**	(0.12–0.53)
Alcohol								
Never	1				1		1	
Occasional	0.71	(0.49–1.02)			0.41*	(0.20–0.83)	0.66	(0.30–1.41)
Regular	0.45**	(0.27–0.75)			0.20**	(0.10–0.47)	0.35	(0.11–1.10)
Physical activity								
No	1				1		1	
Yes	0.23**	(0.14–0.37)			0.07**	(0.02–0.27)	0.15*	(0.03–0.68)

**p < 0.001; *p < 0.05; *OR* Odds Ratio; 95% *CI* Confidence Interval, *CVD* Cardiovascular disease

In step 2 (structural factors models), the variables significantly associated with having diabetes only were age (1.06; 1.04–1.07), Indian (2.93; 1.57–5.49) versus African and secondary education (2.39; 1.18–4.84) versus no education. The variables significantly associated with diabetes and CVD comorbidity were age (1.1; 1.09–1.11), female (2.03; 1.44–2.86) versus males, rural residence (0.59; 0.42–0.81) versus urban residence, being Coloured (1.38; 1.01–1.89) or Indian (2.15; 1.25–3.70) versus being black and primary (2.4; 1.40–4.12), secondary (2.32; 1.29–4.18) or tertiary education (3.52; 1.55–7.99) versus no education. Rural residence was the only variable negatively associated with diabetes-CVD comorbidity implying that those who lived in rural areas were less likely to report this comorbidity.

In step 3 (intermediary factors models), being an occasional smoker (0.04; 0.01–0.22) versus not smoking, being an occasional (0.27; 0.13–0.57) or regular (0.04; 0.01–0.24) alcohol drinker versus not consuming alcohol were the variables associated with having diabetes only. All variables in step 3 were significantly associated with diabetes-CVD comorbidity. A negative relationship was observed for good self-rated health (0.15; 0.07–0.36) versus bad self-rated health, occasional (0.18; 0.06–0.52) and regular (0.21; 0.10–0.46) smokers versus non-smokers, occasional (0.41; 0.20–0.83) and regular (0.20; 0.10–0.47) alcohol drinkers versus not consuming alcohol and physical activity (0.07; 0.02–0.27). This pattern was similar to that observed in the univariate model for the comorbidity studied.

In step 4, the full model, after adjusting for both structural and intermediary factors, age (1.06; 1.03–1.08), being Indian (3.49; 1.11–10.96) versus being African occasional smokers (0.03; 0.00–0.26) versus non-smokers and occasional (0.31; 0.13–0.76) or regular drinkers (0.05; 0.01–0.36) versus no alcohol consumption were the only variables significantly associated with diabetes only. Except for age and being Indian, all other variables mentioned above were negatively associated with diabetes only. Variables significantly associated with diabetes-CVD comorbidity were age (1.09; 1.06–1.12), high income (0.27; 0.10–0.76) versus low income, moderate (0.33; 0.11–0.96) and good self-rated health (0.24; 0.08–0.68) versus bad self-rated health, occasional (0.29; 0.10–0.88) and regular smokers (0.25; 0.12–0.53) versus non-smokers and physical activity (0.15; 0.03–0.68). Age was the only variable positively associated with diabetes-CVD comorbidity. The adjusted model was used to control for potentially confounding variables. It is partly for this reason that we find a noticeable number of non-significant results in the final model (step 4) in comparison to the adjusted model (step 1).

## Discussion

Previous studies have reported on the elevated risk of comorbidities in diabetic patients [2, 6, 16, 17]. However not much is reported on the association between diabetes CVD comorbidity and various behavioural and social determinants. This study is thus important as it adds to the knowledge and literature on diabetes in South Africa and Sub-Saharan Africa in general. Our study finds that high blood pressure, heart disease and stroke were more prevalent amongst diabetic individuals compared to non-diabetics. This finding supports the assertion that the risk of CVDs in diabetics is more than double that of individuals without diabetes [6]. Similar to other studies, we find that high blood pressure was the most common comorbid CVD condition among diabetic individuals, followed by heart disease and stroke [16]. Comorbidity of diabetes and high blood pressure has been found to be high in previous studies [17–19]. This is of great concern given the increase in costs associated with the management of complicated diabetes.

The estimates of diabetes CVD presented provide only a snapshot of the complications commonly reported in diabetic individuals. In our study both diabetes and diabetes CVD comorbidity is more prevalent among individuals with higher education levels (secondary and tertiary). This finding is contrary to that observed in European countries where diabetes is more prevalent among the lower education groups [20]. It should be noted however that the data observed in South Africa may not be directly comparable to other countries because the data presented was self-reported and might be subject to reporting bias. As the symptoms of diabetes are not always correctly interpreted, the self-reported incidences may underreport the true prevalence of diabetes. Also given that the met need for diabetes diagnosis increases with education [21] it is also possible that less educated individuals have not yet been diagnosed. Consistent with other studies we find that diabetes and diabetes CVD comorbidity was more common in those who reside in urban areas [7]. This is perhaps a result of rapid urbanisation, sedentary lifestyles and poor diets.

Strong associations were observed for the structural variables of age, race and income. Our findings are consistent with literature that found that the occurrence of two or more chronic illnesses is associated with age [13, 16, 22]. Diabetes and its comorbid conditions is shown to be very common amongst the elderly [23, 24]. In our study, the mean age of having diabetes and CVD comorbidity was 48 years and 57 years, respectively. Although the occurrence of two or more chronic illnesses is common amongst the elderly, recent studies have shown that it is not only limited to the elderly [25, 26]. A positive association was observed between being Indian and having diabetes, even after adjusting for other variables. This association was however not observed in individuals with diabetes and CVD comorbidity. This relationship has also been observed and discussed in other reports that find that although Indians have a strong genetic predisposition for diabetes, the prevalence of diabetes complications is not associated with any race in particular [7]. Our finding that income is not associated with having diabetes only (without CVDs) is consistent with a South African population based cohort study that found no significant association between diabetes and income [27]. This finding is contrary to that presented in a global systematic review which finds that individuals in low income groups are at an increased risk of diabetes [28]. Whilst the authors find this relationship to be consistently strong in high income countries, they do not find a consistently strong relationship in low and middle income countries and call for further investigations [28]. Our data however indicates that persons with high income were less likely to report having diabetes-CVD comorbidity when compared to individuals with low income. High income could reflect better access and use of healthcare facilities and adoption of good diabetes management practices. This finding warrants further investigation into the income differentials and the effects of socio-economic gradients in diabetes CVD comorbidity.

Various intermediary factors were also found to be associated with either diabetes only or diabetes CVD comorbidity. Empirical studies indicate that exercise interventions are associated with decreases in the incidence of diabetes [29] and that physical inactivity is associated with diabetes [30]. However, our study finds an insignificant association between diabetes and physical activity. The differences in the nature of association between diabetes and physical activity could be a result of variations in definitions and measurements of physical activity. Our study included leisure time physical activity whilst other studies include both leisure time and occupation physical activity [30, 31]. Data on physical activity was collected via patient interview and is therefore subject to social desirability bias. Therefore our estimates may lack precision. However we do find that physical activity is negatively associated with diabetes-CVD comorbidity, implying that those who took part in physical activity were less likely to report diabetes and CVDs comorbidity when compared to those who did not take part in any physical activity. This result is consistent with studies that find that physical activity is associated with reduced risk of CVDs in diabetic individuals [31]. Smoking is also a well-known risk factor of NCDs such as diabetes [11, 16]. In our study surprisingly, being an occasional or regular smoker was negatively associated with diabetes-CVD comorbidity. Using the South African National Income Dynamics Survey data, Alaba and Chola (2013) also find that smoking is negatively associated with the occurrence of two or more chronic conditions in an individual [13]. This unexpected finding calls for further investigations. We however note that caution needs to be observed when interpreting this finding because data on smoking was self-reported and may be subject to social desirability bias. As expected, we found that those who reported moderate and good self-rated health were less likely to report diabetes-CVD comorbidity. This is in line with other studies which found that the presence of multiple chronic illnesses is associated with poor perceived health [32].

Diabetes and CVD comorbidity presents a major challenge to healthcare management and has the potential to increase costs. This study, within its limitations, strengthens the evidence base on the magnitude of diabetes and its comorbid CVD conditions. The identification of the factors associated with diabetes and its comorbid CVD conditions provides useful information that can be used for policy refinement. For example, the finding that high income is negatively associated with diabetes-CVD comorbidity might point to the fact that addressing income inequalities may be essential to reducing diabetes-CVD comorbidity. Encouraging physical activity in diabetic patients may also help in reducing diabetes-CVD comorbidity.

Notwithstanding, there is still a need for a better understanding of the underlying causes of comorbidities between diabetes and other chronic diseases such as neuropathy and nephropathy. The South African National Department of Health strategic plan for the prevention and control of NCDs emphasises the need to strengthen health systems by ensuring that chronic disease management is integrated [11]. The plan sets out to reduce the risk factors associated with NCDs and also increase the percentage of people controlled for diabetes by 2030. In order to influence the plan and or policy, further research into the patterns and implications of diabetes and chronic communicable disease comorbidity is imperative. While our study does not address issues of communicable diseases among persons with diabetes, we identify other common chronic comorbid conditions which should be taken into consideration when designing interventions aimed at integrated management of chronic diseases.

Follow up surveys are crucial in monitoring trends and patterns of diabetes comorbidity and will form an ideal conduit for further research and policy refinement. Given the need for fiscal prudence that characterises the South African economy, addressing diabetes and its comorbid conditions through the targeting of individuals with multiple risk factors is crucial.

### Study strengths and limitations

This study used nationally representative data to determine the prevalence of diabetes-CVD comorbidity and examine the factors associated with this comorbidity at the time of the survey. The study considered three potential diabetes-CVD comorbid conditions that were reported in the dataset, heart disease, high blood pressure and stroke. However, diabetes may be associated with various other comorbid conditions such as depression, osteoarthritis, neuropathy, nephropathy and HIV. We were unable to assess total comorbidity. It is possible that a diabetic individual might have been misclassified as having no other illnesses when in actual fact they were diagnosed with other conditions not reported in the data. This limitation is also exacerbated by the fact that the presence of chronic disease was self-reported. It is possible that some individuals suffered from undiagnosed chronic illnesses resulting in further underreporting. The lack of population level data on diabetes unmet care was reported in one recent study by Manne-Goehler et al. (2016), which revealed both a high prevalence and a large unmet need for diabetes care within sub-Saharan Africa [21]. It is possible that the prevalence of diabetes was underestimated and that there is a high unmet need for diabetes care in South Africa. Because diabetes was self-reported, we were also unable to classify the type of diabetes that was reported therefore our study included risk factors such as physical inactivity common to type 2 diabetes. It is important to note that the data used is this study is self-reported and is subject to social desirability bias. Due to the cross-sectional nature of the study, casual inferences could not be drawn.

## Conclusion

To our knowledge, this is the first published study in South Africa to look at diabetes-CVD comorbidity and its association with various socio-economic, behavioural and demographic factors. Diabetes and its comorbid conditions potentially place a heavy burden on the healthcare system as a result of increased demand for healthcare. Studies like ours contribute to the current pool of knowledge about factors predisposing, promoting and establishing disease in patients with previously existing illness [33]. Our study highlighted differences in the correlates of diabetes and CVD comorbidity. High income and physical activity were significantly associated with diabetes-CVD comorbidity. Evidence has shown that lifestyle habits such as physical activity are essential for the management of diabetes [2]. In our study, we also show that the elderly are at risk of having diabetes or diabetes-CVD comorbidity. Given the rapidly increasing size of the older population, it is critical to generate evidence on the magnitude of diabetic comorbidity in South Africa in order to inform the development of interventions for the prevention of diabetic comorbidity and strengthen the healthcare system. Health sector policy reforms such as the National Health Insurance [33] should target vulnerable groups such as the elderly as they are at risk of chronic illnesses. The monitoring of diabetic comorbidity will guide research into the capabilities of primary healthcare workers in holistically managing comorbidity. Our study consistently showed that those who took part in any physical activity were less likely to report diabetes-CVD comorbidity. There is a need to put in place measures and interventions that encourage more active and healthy lifestyles.

## Abbreviations

BMI: Body Mass Index
CI: Confidence Interval
CSDH: Commission on Social Determinants of Health
CVD: Cardio-vascular disease
EA: Enumeration Area
HSRC: Human Sciences Research Council
LMIC: Low- and Middle-Income Countries
NCD: Non Communicable Disease
OR: Odds Ratio
REC: Research Ethics Committee
SANHANES-1: South African National Health and Nutrition Examination Survey
SD: Standard Deviation
VP: Visiting Point
